# *N*,*N*,2,4,6-Penta­methyl­anilinium hexa­fluoro­phosphate

**DOI:** 10.1107/S1600536812049379

**Published:** 2012-12-08

**Authors:** Yuan Zhang

**Affiliations:** a Department of Applied Chemistry, Nanjing College of Chemical Technology, Nanjing 210048, People’s Republic of China

## Abstract

In the crystal structure of the title salt, C_11_H_18_N^+^·PF_6_^−^, the cation and anion are connected *via* an N—H⋯F hydrogen bond; weak C—H⋯F hydrogen bonding also occurs between the cations and anions.

## Related literature

For the background to the title salt, see: Haertling *et al.* (1999 ▶); Homes *et al.* (2001 ▶).
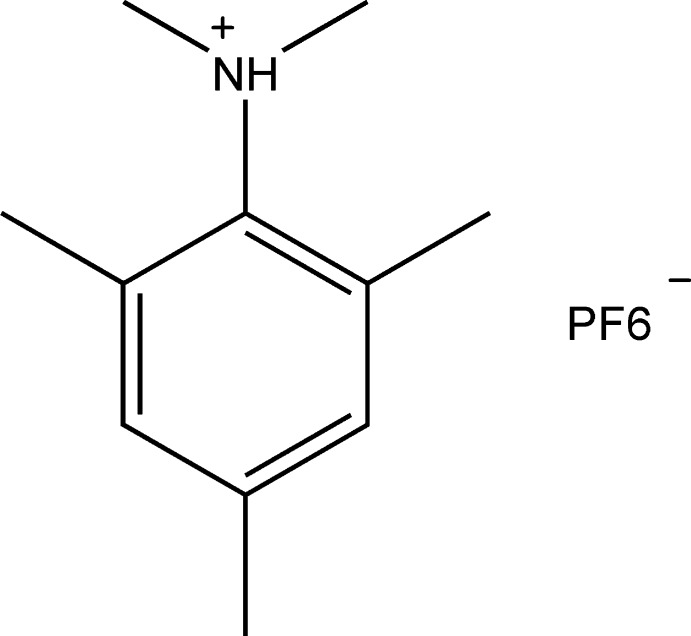


## Experimental

### 

#### Crystal data

C_11_H_18_N^+^·PF_6_^−^
*M* *_r_* = 309.23
Monoclinic, 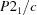

*a* = 11.466 (2) Å
*b* = 8.2439 (16) Å
*c* = 15.559 (3) Å
β = 97.82 (3)°
*V* = 1457.0 (5) Å^3^
*Z* = 4
Mo *K*α radiation
μ = 0.24 mm^−1^
*T* = 293 K
0.20 × 0.19 × 0.18 mm

#### Data collection

Rigaku Mercury2 diffractometer
14679 measured reflections
3340 independent reflections
1942 reflections with *I* > 2σ(*I*)
*R*_int_ = 0.074

#### Refinement

*R*[*F* ^2^ > 2σ(*F* ^2^)] = 0.063
*wR*(*F* ^2^) = 0.186
*S* = 1.03
3340 reflections
177 parameters
H-atom parameters constrained
Δρ_max_ = 0.21 e Å^−3^
Δρ_min_ = −0.27 e Å^−3^

### 

Data collection: *CrystalClear* (Rigaku, 2005 ▶); cell refinement: *CrystalClear*; data reduction: *CrystalClear*; program(s) used to solve structure: *SHELXTL* (Sheldrick, 2008 ▶); program(s) used to refine structure: *SHELXTL*; molecular graphics: *SHELXTL*; software used to prepare material for publication: *SHELXTL*.

## Supplementary Material

Crystal structure: contains datablock(s) I, global. DOI: 10.1107/S1600536812049379/xu5658sup1.cif

Structure factors: contains datablock(s) I. DOI: 10.1107/S1600536812049379/xu5658Isup2.hkl

Supplementary material file. DOI: 10.1107/S1600536812049379/xu5658Isup3.cml

Additional supplementary materials:  crystallographic information; 3D view; checkCIF report

## Figures and Tables

**Table 1:** Hydrogen-bond geometry (Å, °)

*D*—H⋯*A*	*D*—H	H⋯*A*	*D*⋯*A*	*D*—H⋯*A*
N1—H1⋯F1	0.91	2.27	2.979 (3)	134
C11—H11*A*⋯F4^i^	0.96	2.47	3.399 (4)	162

Symmetry code: (i) 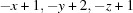.

**Fig. 1.**
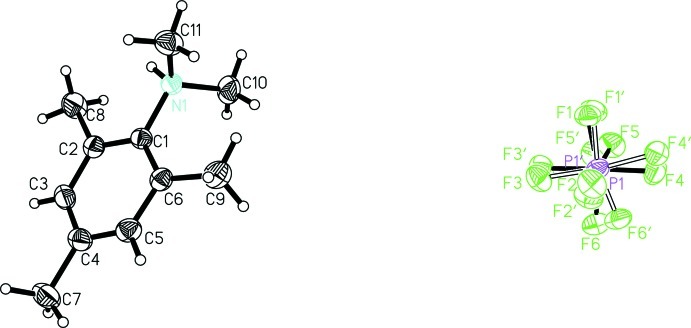
Perspective structure of the title compound, showing the atom-numbering scheme. Displacement ellipsoids are drawn at the 30% probability level.

**Fig. 2.**
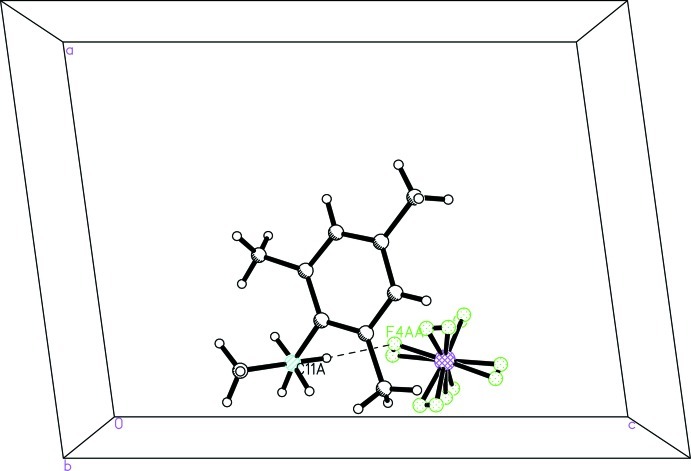
The crystal packing of the title compound viewed along theb axis showing the hydrogen bondings network.

## supplementary crystallographic information

### Comment

At present, much attention in ferroelectric material field is focused on developing ferroelectric pure organic or inorganic compounds (Haertling *et al.* 1999; Homes *et al.* 2001). In order to find more dielectric ferroelectric materials, we investigate the physical properties of the title compound (Fig. 1). The dielectric constant of the title compound as a function of temperature indicates that the permittivity is basically temperature-independent (dielectric constant equaling to 3.7 to 5.2), suggesting that this compound should be not a real ferroelectrics or there may be no distinct phase transition occurred within the measured temperature range. Similarly, below the melting point (453 K) of the compound, the dielectric constant as a function of temperature also goes smoothly, and there is no dielectric anomaly observed (dielectric constant equaling to 3.7 to 5.2). Herein, we report the synthesis and crystal structure of the title compound.

Molecules of the title compound have normal geometric parameters. The bond lengths and angles are within their normal ranges. In the crystal, the cation and anion are connected *via* N—H···F and weak C—H···F hydrogen bonds (Table 1).

### Experimental

A mix of *N*,*N*,2,4,6-pentamethylbenzenamine (1.36 g, 0.01 mol) and hexafluorophosphoric acid (1.90 g, 0.01 mol) in methanol (20 ml) was stirred until clear. After several days, the title compound was formed and recrystallized from methanol solution to afford colourless prismatic crystals suitable for X-ray analysis.

### Refinement

H atoms were positioned geometrically and refined using a riding model, with C—H = 0.93–0.96 and N—H = 0.91 Å and *U*_iso_(H) = 1.2_eq_(C,N).

### Crystal data

**Table:**

C_11_H_18_N^+^·PF_6_^−^	*F*(000) = 640
*M**_r_* = 309.23	*D*_x_ = 1.410 Mg m^−^^3^
Monoclinic, *P*2_1_/*c*	Mo *K*α radiation, λ = 0.71073 Å
Hall symbol: -P 2ybc	Cell parameters from 3340 reflections
*a* = 11.466 (2) Å	θ = 2.7–27.5°
*b* = 8.2439 (16) Å	µ = 0.24 mm^−^^1^
*c* = 15.559 (3) Å	*T* = 293 K
β = 97.82 (3)°	Prism, colorless
*V* = 1457.0 (5) Å^3^	0.20 × 0.19 × 0.18 mm
*Z* = 4	

### Data collection

**Table:**

Rigaku Mercury2 diffractometer	1942 reflections with *I* > 2σ(*I*)
Radiation source: fine-focus sealed tube	*R*_int_ = 0.074
Graphite monochromator	θ_max_ = 27.5°, θ_min_ = 3.1°
Detector resolution: 13.6612 pixels mm^-1^	*h* = −14→14
CCD_Profile_fitting scans	*k* = −10→10
14679 measured reflections	*l* = −20→20
3340 independent reflections	

### Refinement

**Table:**

Refinement on *F*^2^	Primary atom site location: structure-invariant direct methods
Least-squares matrix: full	Secondary atom site location: difference Fourier map
*R*[*F*^2^ > 2σ(*F*^2^)] = 0.063	Hydrogen site location: inferred from neighbouring sites
*wR*(*F*^2^) = 0.186	H-atom parameters constrained
*S* = 1.03	*w* = 1/[σ^2^(*F*_o_^2^) + (0.0788*P*)^2^ + 0.4571*P*] where *P* = (*F*_o_^2^ + 2*F*_c_^2^)/3
3340 reflections	(Δ/σ)_max_ = 0.001
177 parameters	Δρ_max_ = 0.21 e Å^−^^3^
0 restraints	Δρ_min_ = −0.27 e Å^−^^3^

### Special details

**Table:**

Geometry. All esds (except the esd in the dihedral angle between two l.s. planes) are estimated using the full covariance matrix. The cell esds are taken into account individually in the estimation of esds in distances, angles and torsion angles; correlations between esds in cell parameters are only used when they are defined by crystal symmetry. An approximate (isotropic) treatment of cell esds is used for estimating esds involving l.s. planes.
Refinement. Refinement of F^2^ against ALL reflections. The weighted R-factor wR and goodness of fit S are based on F^2^, conventional R-factors R are based on F, with F set to zero for negative F^2^. The threshold expression of F^2^ > 2sigma(F^2^) is used only for calculating R-factors(gt) etc. and is not relevant to the choice of reflections for refinement. R-factors based on F^2^ are statistically about twice as large as those based on F, and R- factors based on ALL data will be even larger.

### Fractional atomic coordinates and isotropic or equivalent isotropic displacement parameters (Å^2^)

**Table:**

	*x*	*y*	*z*	*U*_iso_*/*U*_eq_	
P1	0.32971 (7)	0.80605 (11)	0.35021 (6)	0.0588 (3)	
N1	0.68536 (19)	0.8547 (3)	0.37739 (14)	0.0466 (6)	
H1	0.6213	0.8201	0.4009	0.056*	
F1	0.43410 (18)	0.9123 (3)	0.39786 (18)	0.1138 (9)	
F2	0.2284 (2)	0.6948 (3)	0.30436 (15)	0.0956 (8)	
F3	0.3564 (2)	0.8663 (3)	0.25858 (15)	0.1075 (8)	
F4	0.3043 (2)	0.7382 (4)	0.44062 (14)	0.1104 (9)	
F5	0.4216 (2)	0.6632 (3)	0.34402 (15)	0.0948 (7)	
F6	0.2418 (2)	0.9486 (3)	0.3564 (2)	0.1270 (11)	
C1	0.7884 (2)	0.7941 (3)	0.43803 (17)	0.0432 (6)	
C2	0.7626 (2)	0.7091 (3)	0.51013 (18)	0.0482 (7)	
C3	0.8564 (3)	0.6512 (4)	0.56766 (19)	0.0559 (8)	
H3	0.8409	0.5922	0.6158	0.067*	
C4	0.9722 (3)	0.6782 (4)	0.5560 (2)	0.0560 (8)	
C5	0.9930 (3)	0.7638 (4)	0.4838 (2)	0.0568 (8)	
H5	1.0706	0.7832	0.4757	0.068*	
C6	0.9034 (2)	0.8228 (3)	0.42212 (18)	0.0487 (7)	
C7	1.0726 (3)	0.6148 (4)	0.6206 (2)	0.0763 (11)	
H7A	1.1392	0.5919	0.5912	0.114*	
H7B	1.0484	0.5172	0.6468	0.114*	
H7C	1.0940	0.6949	0.6647	0.114*	
C8	0.6383 (3)	0.6771 (5)	0.5279 (2)	0.0735 (10)	
H8A	0.6403	0.6149	0.5802	0.110*	
H8B	0.5964	0.6177	0.4803	0.110*	
H8C	0.5991	0.7784	0.5344	0.110*	
C9	0.9394 (3)	0.9078 (5)	0.3437 (2)	0.0707 (10)	
H9A	0.9402	0.8312	0.2973	0.106*	
H9B	1.0166	0.9534	0.3584	0.106*	
H9C	0.8842	0.9928	0.3256	0.106*	
C10	0.6719 (3)	0.7799 (5)	0.2888 (2)	0.0751 (10)	
H10A	0.7327	0.8200	0.2576	0.113*	
H10B	0.5963	0.8079	0.2580	0.113*	
H10C	0.6783	0.6642	0.2940	0.113*	
C11	0.6748 (3)	1.0353 (4)	0.3754 (2)	0.0693 (9)	
H11A	0.6797	1.0758	0.4336	0.104*	
H11B	0.6005	1.0656	0.3434	0.104*	
H11C	0.7375	1.0806	0.3480	0.104*	

### Atomic displacement parameters (Å^2^)

**Table:**

	*U*^11^	*U*^22^	*U*^33^	*U*^12^	*U*^13^	*U*^23^
P1	0.0438 (5)	0.0666 (6)	0.0658 (6)	−0.0018 (4)	0.0060 (4)	−0.0077 (4)
N1	0.0410 (12)	0.0534 (14)	0.0461 (13)	−0.0011 (10)	0.0077 (10)	0.0047 (10)
F1	0.0631 (13)	0.121 (2)	0.154 (2)	−0.0277 (14)	0.0021 (14)	−0.0462 (17)
F2	0.0882 (15)	0.1006 (17)	0.0926 (16)	−0.0308 (13)	−0.0073 (12)	−0.0137 (12)
F3	0.126 (2)	0.1015 (18)	0.0994 (18)	0.0058 (15)	0.0303 (15)	0.0358 (14)
F4	0.1209 (19)	0.148 (2)	0.0674 (15)	−0.0243 (18)	0.0314 (13)	−0.0149 (14)
F5	0.0918 (15)	0.0934 (16)	0.1004 (17)	0.0358 (13)	0.0172 (13)	0.0080 (12)
F6	0.0688 (14)	0.0922 (18)	0.222 (3)	0.0201 (13)	0.0262 (16)	−0.0326 (19)
C1	0.0427 (14)	0.0416 (15)	0.0451 (15)	0.0000 (12)	0.0057 (11)	−0.0008 (12)
C2	0.0487 (15)	0.0503 (17)	0.0466 (16)	0.0005 (13)	0.0096 (12)	0.0003 (13)
C3	0.064 (2)	0.0586 (19)	0.0435 (16)	0.0016 (15)	0.0034 (14)	0.0015 (13)
C4	0.0576 (18)	0.0516 (18)	0.0548 (18)	0.0065 (14)	−0.0069 (14)	−0.0121 (14)
C5	0.0431 (15)	0.0622 (19)	0.065 (2)	−0.0031 (14)	0.0062 (14)	−0.0076 (16)
C6	0.0448 (15)	0.0517 (17)	0.0504 (17)	−0.0029 (13)	0.0092 (12)	−0.0023 (13)
C7	0.076 (2)	0.074 (2)	0.070 (2)	0.0143 (19)	−0.0217 (18)	−0.0071 (18)
C8	0.059 (2)	0.096 (3)	0.068 (2)	−0.0054 (18)	0.0193 (16)	0.0252 (19)
C9	0.0540 (18)	0.086 (2)	0.076 (2)	−0.0051 (18)	0.0245 (16)	0.0151 (19)
C10	0.067 (2)	0.105 (3)	0.051 (2)	−0.005 (2)	−0.0001 (15)	−0.0204 (18)
C11	0.069 (2)	0.060 (2)	0.079 (2)	0.0075 (17)	0.0088 (17)	0.0133 (17)

### Geometric parameters (Å, º)

**Table:**

P1—F6	1.560 (2)	C5—C6	1.394 (4)
P1—F2	1.573 (2)	C5—H5	0.9300
P1—F3	1.579 (2)	C6—C9	1.512 (4)
P1—F4	1.577 (3)	C7—H7A	0.9600
P1—F1	1.584 (2)	C7—H7B	0.9600
P1—F5	1.591 (2)	C7—H7C	0.9600
N1—C11	1.494 (4)	C8—H8A	0.9600
N1—C1	1.494 (3)	C8—H8B	0.9600
N1—C10	1.499 (4)	C8—H8C	0.9600
N1—H1	0.9100	C9—H9A	0.9600
C1—C2	1.388 (4)	C9—H9B	0.9600
C1—C6	1.394 (4)	C9—H9C	0.9600
C2—C3	1.387 (4)	C10—H10A	0.9600
C2—C8	1.512 (4)	C10—H10B	0.9600
C3—C4	1.382 (4)	C10—H10C	0.9600
C3—H3	0.9300	C11—H11A	0.9600
C4—C5	1.374 (4)	C11—H11B	0.9600
C4—C7	1.515 (4)	C11—H11C	0.9600
			
F6—P1—F2	91.36 (14)	C6—C5—H5	118.4
F6—P1—F3	91.42 (16)	C1—C6—C5	116.4 (3)
F2—P1—F3	89.77 (14)	C1—C6—C9	126.2 (3)
F6—P1—F4	90.74 (17)	C5—C6—C9	117.4 (3)
F2—P1—F4	89.02 (14)	C4—C7—H7A	109.5
F3—P1—F4	177.55 (15)	C4—C7—H7B	109.5
F6—P1—F1	90.47 (14)	H7A—C7—H7B	109.5
F2—P1—F1	177.89 (15)	C4—C7—H7C	109.5
F3—P1—F1	91.24 (15)	H7A—C7—H7C	109.5
F4—P1—F1	89.91 (15)	H7B—C7—H7C	109.5
F6—P1—F5	178.83 (14)	C2—C8—H8A	109.5
F2—P1—F5	89.71 (14)	C2—C8—H8B	109.5
F3—P1—F5	88.11 (13)	H8A—C8—H8B	109.5
F4—P1—F5	89.76 (15)	C2—C8—H8C	109.5
F1—P1—F5	88.47 (13)	H8A—C8—H8C	109.5
C11—N1—C1	113.6 (2)	H8B—C8—H8C	109.5
C11—N1—C10	113.1 (3)	C6—C9—H9A	109.5
C1—N1—C10	114.6 (2)	C6—C9—H9B	109.5
C11—N1—H1	104.7	H9A—C9—H9B	109.5
C1—N1—H1	104.7	C6—C9—H9C	109.5
C10—N1—H1	104.7	H9A—C9—H9C	109.5
C2—C1—C6	122.7 (2)	H9B—C9—H9C	109.5
C2—C1—N1	116.2 (2)	N1—C10—H10A	109.5
C6—C1—N1	121.1 (2)	N1—C10—H10B	109.5
C1—C2—C3	117.6 (3)	H10A—C10—H10B	109.5
C1—C2—C8	123.0 (3)	N1—C10—H10C	109.5
C3—C2—C8	119.3 (3)	H10A—C10—H10C	109.5
C4—C3—C2	122.3 (3)	H10B—C10—H10C	109.5
C4—C3—H3	118.9	N1—C11—H11A	109.5
C2—C3—H3	118.9	N1—C11—H11B	109.5
C5—C4—C3	117.8 (3)	H11A—C11—H11B	109.5
C5—C4—C7	121.3 (3)	N1—C11—H11C	109.5
C3—C4—C7	120.9 (3)	H11A—C11—H11C	109.5
C4—C5—C6	123.2 (3)	H11B—C11—H11C	109.5
C4—C5—H5	118.4		

### Hydrogen-bond geometry (Å, º)

**Table:**

*D*—H···*A*	*D*—H	H···*A*	*D*···*A*	*D*—H···*A*
N1—H1···F1	0.91	2.27	2.979 (3)	134
C11—H11*A*···F4^i^	0.96	2.47	3.399 (4)	162

Symmetry code: (i) −*x*+1, −*y*+2, −*z*+1.
